# Plant Cyclin-Dependent Kinase Inhibitors of the KRP Family: Potent Inhibitors of Root-Knot Nematode Feeding Sites in Plant Roots

**DOI:** 10.3389/fpls.2017.01514

**Published:** 2017-09-08

**Authors:** Paulo Vieira, Janice de Almeida Engler

**Affiliations:** ^1^Laboratório de Nematologia, Instituto de Ciências Agrárias e Ambientais Mediterrânicas, Universidade de Évora Évora, Portugal; ^2^Institut National de la Recherche Agronomique, Centre National de la Recherche Scientifique, Institut Sophia Agrobiotech, Université Côte d’Azur Nice, France

**Keywords:** cyclin dependent kinase, giant cells, kip-related proteins, *Meloidogyne incognita*, *Arabidopsis thaliana*

## Abstract

Root-knot nematodes (RKN), *Meloidogyne* spp., are distributed worldwide and impose severe economic damage to many agronomically important crops. The plant cell cycle machinery is considered one of the pivotal components for the formation of nematode feeding sites (NFSs) or galls. These feeding sites contain five to nine hypertrophied giant cells (GC) resulting from developmental reprogramming of host root cells by this pathogen. GC undergo synchronous waves of mitotic activity uncoupled from cytokinesis giving rise to large multinucleate cells. As development of the NFS progresses, multiple rounds of DNA synthesis occur in the nuclei of GC, coupled with nuclear and cellular expansion. These cells are highly metabolically active and provide the nematode with nutrients necessary for its development and completion of its life cycle. In *Arabidopsis* seven cyclin dependent kinase inhibitors (CKIs) belonging to the interactors/inhibitors of the cyclin dependent kinases (ICK) family, also referred as Kip-Related Proteins (KRPs) have been identified. Interactions of KRPs with CDK/Cyclin complexes decrease CDK activity, affecting both cell cycle progression and DNA content in a concentration-dependent manner. We performed the functional analysis of all *Arabidopsis KRP* gene members during RKN interaction in *Arabidopsis* to obtain more insight into their role during gall development. We demonstrated that three members of this family (*KRP2*, *KRP5*, and *KRP6*) were highly expressed in galls and were important for cell cycle regulation during NFS development as shown by their different modes of action. We also pointed out that cell cycle inhibition through overexpression of all members of the KRP family can affect NFS development and consequently compromise the nematode’s life cycle. In this review we summarized our recent understanding of the KRP family of genes, and their role in controlling cell cycle progression at the RKN feeding site.

## Introduction

A large number of crop species worldwide are harmed by plant-parasitic nematodes like root-knot nematodes (RKN), namely *Meloidogyne* spp. In the case of RKN, roots of the host plants are invaded by the motile, infective second-stage juveniles (J2), which induce dramatic changes in particular to the root vascular cells, ultimately producing a complex nematode feeding site (NFS) (Jones and Northcote, 1972). These sophisticated changes that occur in root cells during nematode infection, involve the formation of root swellings named galls, which contain the giant cells (GC) that are the nematode feeding cells. GC are the main source of nutrients that allow development and reproduction of this parasitic nematode. Parallel to GC development, a network of surrounding neighboring cells (NC) divides asymmetrically, ultimately supporting the transfer of nutrients into the GC (Hoth et al., 2008; Rodiuc et al., 2014). Together GC and NC constitute the complete NFS, which surrounded by cortical and epidermal cells, is apparent in the host root as the typical root-knot or gall.

The large number of host genes involved demonstrates the complexity of the plant–RKN interaction. Transcriptional data showed an extensive regulation of various host molecular pathways, most likely through the crosstalk between the nematode-secreted proteins and their host molecular targets (Gheysen and Mitchum, 2009). Although the molecular mechanisms behind the formation and development of the gall are still far from being completely understood, the activation and regulation of the host cell cycle machinery by RKN has been confirmed to be an essential process leading to the formation of multinucleated GC and gall expansion (de Almeida Engler et al., 2015).

The apparently balanced cell cycle gene expression occurring during the NFS development is characterized by two major cell cycle mechanisms. First, a recurring synchronized mitotic phase uncoupled from cytokinesis that lasts around 10 days in *Arabidopsis*, drives the formation of the multinucleate state of each giant cell followed by endoreduplication. Multiple rounds of DNA synthesis lacking mitosis accompany GC expansion and nuclei enlargement with a corresponding increase in ploidy level (de Almeida Engler et al., 1999, 2012). During both activation and progression of the cell cycle in NFS, the nematode most likely regulates the formation of the gall by its secreted proteins, and also acquires nutrients from the GC that are required for their development and maturation. RKN development comprises distinct juvenile developmental stages (J1 to J4) up to the formation of the typical egg laying, pear-shaped female, followed by offspring production. Distinct cellular features of these highly metabolically active GC are dense cytoplasm filled with profuse organelles, small vacuoles and cell wall ingrowths (Rodiuc et al., 2014).

The basic control mechanisms that regulate progression through the cell cycle are remarkably well conserved among eukaryotes. The cell cycle in plant cells is controlled by highly conserved complexes formed by cyclin-dependent kinases (CDKs) with their regulatory cyclin subunits (CYCs), which are needed for ensuring correct temporal and unidirectional progression of the cell cycle phases (Inzé and De Veylder, 2006). Expression of cell cycle genes in the plant host appears tightly regulated as the gall matures, implying a strict control of the cell cycle machinery and its molecular components during the plant–nematode interaction (de Almeida Engler et al., 2015). Cell cycle genes identified to date that participate in the ontogeny of RKN-induced galls have been extensively reviewed elsewhere (de Almeida Engler and Gheysen, 2013; de Almeida Engler et al., 2015). Herein, we focus on providing a summarized and global assessment of our recent understanding of a particular family of cyclin-dependent kinase inhibitor (*CKI*) genes that regulate cell cycle progression in the RKN feeding site using *Arabidopsis* as a model host.

In *Arabidopsis* seven CDK inhibitors belonging to the interactors/inhibitors of CDK (ICK) family, also referred to as Kip-Related Proteins (KRPs), have been identified (Wang et al., 1997; De Veylder et al., 2001). The KRPs are small proteins with a C-terminal domain required for CDK- or cyclin-binding, and they function as inhibitors (De Veylder et al., 2001; Inzé and De Veylder, 2006). They have different spatial expression and distinct temporal and functional patterns (De Veylder et al., 2001; Menges and Murray, 2002; Ormenese et al., 2004; Menges et al., 2005; Verkest et al., 2005a; Wang et al., 2006). This family of genes is known to bind to CDKA-CYCD complexes (Wang et al., 2006; Van Leene et al., 2010). The fine-tuning of KRP protein levels in plant cells is a key factor to maintain the balance between cell proliferation and cell differentiation (Verkest et al., 2005b; Inzé and De Veylder, 2006). Interactions of KRPs and CDK/CYCs complexes reduce CDK activity, and can influence both cell cycle progression and DNA replication in a concentration-dependent manner (Verkest et al., 2005b). Because RKN infection leads to the formation of multinucleate GC involving recurrent activity of the host cell cycle machinery, the specific involvement of the complete *KRP* gene family has been investigated. Our studies revealed that these cell cycle inhibitors exert distinct functions in a RKN-induced feeding site. Transcriptional activity and functional studies of *KRP2*, *KRP5* and *KRP6* revealed their participation in the regulation of the cell cycle machinery implicated in gall formation and expansion. Although *KRP1*, *KRP3*, *KRP4* and *KRP7* are not expressed in galls, their ectopic expression inhibited gall development and nematode maturation. Herein, we focused on major results obtained during our functional analyses (summarized in **Table 1**) of this gene family and briefly discuss their potential use for biotechnology applications.

**Table 1:** Summarized overview of functional characterization studies of the *Arabidopsis* Kip-Related Protein (KRP) family in galls induced by the root-knot nematode *Meloidogyne incognita.*

	Uninfected roots						Knockout (KO) KRP lines			Overexpression (OE) KRP Lines					
Gene	Promoter activity			GFP localization			Gall phenotype		Resistant test	Gall phenotype			Nuclei phenotype	Ploidy levels	Resistant test
	VC	GC	NC	RAM	GC	NC	GC	NC		GC		NC	GC		
*KRP1*	-	-	-	Nucleus and sub-nuclear dots			NA	NA	NA	↓ Number of nuclei and giant cell size	↓	↓ Number of NC	Elongated and grouped	↑ GC ploidy levels	↓ Number of Galls ↓ Number of egg masses
*KRP2*	+	+	+	Nucleus			↑ Number of nuclei ↑ Mitotic figures	↑ Number of NC	Elongated and grouped= to wild-type	↓ Number of nuclei and giant cell size	↓	↓ Number of NC	= to wild-type	↓ GC ploidy levels	↓ Number of Galls ↓ Number of egg masses
*KRP3*	-	-	-	Nucleus and sub-nuclear dots			NA	NA	NA	↓ Number of nuclei and giant cell size, with elongated shape	↓	↓ Number of NC		↑ GC ploidy levels	↓ Number of Galls ↓ Number of egg masses
*KRP4*	-	-	-	Nucleus and sub-nuclear dots			NA	NA	NA	↓ Number of nuclei and giant cell size	↓	↓ Number of NC	Elongated and grouped	NP	↓ Number of egg masses
*KRP5*	+	+	+	Nucleus and sub-nuclear dots			↓Cytoplasm content ↑ Larger vacuoles	= to wild-type	↓ Number of egg masses	= to wild-type		↓ Number of NC	Elongated and grouped	= to wild-type	↓ Number of Galls ↓ Number of egg masses
*KRP6*	+	+	+	Nucleus			↑Cell wall stubs ↓Giant cell size	= to wild-type	= to wild-type	↑ Number of nuclei ↓ Giant cell size	↓	↑ Number of NC	= to wild-type	↓ GC ploidy levels	↓ Number of egg masses
*KRP7*	-	-	-	Nucleus			NA	NA	NA	↓ Number of nuclei and giant cell size	↓	↓ Number of NC	= to wild-type	↑ GC ploidy levels	↓ Number of Galls ↓ Number of egg masses


*+, expressed. -, not expressed. =, similar. Low arrow, decreased in comparison to wild-type. High arrow, increased in comparison to wild-type. VC, vascular cylinder; GC, Giant cell; NC, Neighboring cell; RAM, root apical meristem; NA, non-applicable; NP, not performed*.

## An Insight of the *Krp* Gene Family During Nematode Feeding Site Development in *Arabidopsis*

Promoter activity as well as expression analysis of the seven *Arabidopsis KRP* genes (*KRP1* to *KRP7*) have been examined in detail in uninfected roots and during several stages of gall development (Vieira et al., 2012, 2013, 2014; Coelho et al., 2017). Our data illustrated that three out of the seven members of the *KRP* gene family, namely *KRP2*, *KRP5* and *KRP6*, are expressed in RKN induced galls and present during a specific time span of the NFS development (**Table 1**). *KRP2*, *KRP5* and *KRP6* transcripts were detected during early gall development [∼7 days after inoculation (DAI)] in agreement with the high mitotic activity occurring in the GC. This suggests that a level of cell cycle regulation must be triggered in order to control GC formation. During gall maturation (>14DAI), only *KRP2* and *KRP5* showed a significant transcription activity in GC implying their role in the endoreduplication cycle at this stage of gall development (Vieira et al., 2013, 2014; Coelho et al., 2017). Conversely, the remaining *KRP1*, *KRP3*, *KRP4* and *KRP7* genes were not expressed in the galls at any time point during nematode infection. *KRP* genes (*KRP2*, *KRP5*, and *KRP6*) present in the root vascular of non-infected tissues were also expressed in the galls. Likewise, no particular induction of the remaining *KRP* genes (*KRP1, KRP3, KRP4*, and *KRP7*) was seen in roots upon RKN infection. Therefore, for gall growth and development, *KRP* genes appeared to be regulated and followed their natural location in the root during nematode infection (Vieira et al., 2013).

Kip-Related Proteins are proteins that show a strict nuclear localization during interphase, with some members, namely KRP1, KRP3, KRP4 and KRP5, also displaying a subnuclear localization (Bird et al., 2007). This punctuated pattern has been associated with chromocenters (Jakoby et al., 2006; Bird et al., 2007), which are densely stained regions linked with chromatin (Jégu et al., 2013). Protein localization followed by time-lapse confocal microscopy of uninfected *Arabidopsis* root cells revealed that during mitosis KRP1-GFP, KRP3-GFP, GFP-KRP4, and GFP-KRP5 proteins co-localized with chromosomes (**Table 1**). This localization suggests their activity during interphase, as well as during different stages of mitosis. On the other hand, GFP-KRP2, GFP-KRP6, KRP7-GFP proteins became dispersed into the cytoplasm after nuclear envelope break down during mitosis presenting no apparent co-localization to the chromosomes. These proteins will later uniformly re-accumulate in the nuclei of the two newly formed daughter cells (Vieira et al., 2013, 2014; Coelho et al., 2017).

Our protein localization studies suggest that the dynamic post-translational regulation of the KRP members (KRP2, KRP5 and KRP6) implicated in gall formation might also occur during the RKN–plant interaction. It is still unknown as to whether the post-translational regulation is targeted directly or indirectly by the RKN secreted proteins. Although transcript analyses illustrated continuous detection of *KRP2*, the protein expression profile exposed a weak GFP-KRP2 accumulation in nuclei of young GC (2-14DAI), while in maturing galls (>14DAI) the enhanced accumulation of GFP-KRP2 in the GC nuclei suggested its involvement in endoreduplication control (Vieira et al., 2013). Previously, we had shown a strong *CDKB1;1* promoter activity in young galls (de Almeida Engler et al., 1999). CDKA functions in coordination with B-type CDKs to promote the transition from G2 to mitosis, and KRP2 is considered an important regulator of the G2-M transition in *Arabidopsis* (Inzé and De Veylder, 2006). As KRP2 operates in a dosage-dependent manner, B-type CDKs phosphorylate KRP2 triggering their destruction and allowing cell proliferation. B-type CDK activity ceases in cells triggered to enter endoreduplication resulting in stabilization of KRPs, which bind and inhibit CDK-CYC complexes that have a role in mitosis (Verkest et al., 2005b). Accordingly, the reduced expression levels of *CDKB1;1* at intermediate to advanced GC developmental stages (14–21 DAI) (de Almeida Engler et al., 1999), overlap with the increased labeling of GFP-KRP2 within the GC nuclei (Vieira et al., 2013). Protein profiles suggest that the fluctuations of KRP2 in the GC nuclei may reflect its regulation by CDKB;1 at early time points, allowing mitotic divisions to take place within the GC. At later time points KRP2 accumulation could mediate the switch from a mitotic cell cycle toward the endoreduplication status of the GC.

In the case of KRP5, *in vivo* examination of RKN-infected roots treated with the proteasome inhibitor MG132, showed enhanced GFP-KRP5 accumulation in the GC nuclei suggesting that KRP5 protein levels are potentially controlled by degradation of the 26S proteasome protein (Coelho et al., 2017). Our observations indicated that KRP5 accompanied the process of GC maturation, and this might be important for cell cycle regulation during the NFS expansion (Coelho et al., 2017). *KRP5* has been found to be preferentially expressed in endoreduplicating cells during etiolation and is considered a positive regulator of endoreduplication (Jégu et al., 2013). It has also been shown that KRP5 binds to various loci on chromatin in nuclear localization studies (Jégu et al., 2013). In addition to its CDK/CYC inhibitory capacity, KRP5 has been also implicated in the regulation of transcription, suggesting multifaceted functions for KRP5 that include cell elongation and endoreduplication (Jégu et al., 2013). The fact that KRP5 can target heterochromatin regions suggests that KRP5 is also involved in the modulation of chromatin organization, as its overexpression resulted in an increase of chromocenter decondensation (Jégu et al., 2013). Similar to uninfected plants (Jégu et al., 2013), KRP5 expression could be linked to the expansion of the GC (Coelho et al., 2017).

Detection of GFP-KRP6 within the gall tissues was restricted to the mitotic nuclei of both GC and NC at early stages of gall development (2–10 DAI). As GC development progressed, a decline to absence of GFP-KRP6 expression took place within the GC nuclei, while expression continued in nuclei of the dividing NC (Vieira et al., 2014). This implies that the mitotic phase of GC appears to rely on the presence of proteins like KRP6 that promote mitosis and possibly inhibit cytokinesis (Vieira et al., 2014). The combination of these studies supports the incipient connection between cell cycle activity and the importance of *KRP* genes as regulatory agents with distinct substrates (e.g., different CDK/CYC complexes) and phase-specific functions. Therefore, *KRP*s might be acting and contributing to the sequential progression of the cell cycle in galls.

To determine the potential role of *KRP* genes normally expressed in NFS, loss of function mutants of *KRP2*, *KRP5* and *KRP6* were challenged with RKN. For *krp2* and *krp2^-/-^/krp6^-/-^* mutants, morphological analyses of galls revealed an intensification of the mitotic activity leading to an increased number of nuclei within the GC and proliferation of the surrounding NC (Vieira et al., 2013). This accelerated mitotic activity in the gall was supported by data showing that loss of function of *krp*2 increased the rate of lateral root formation (Sanz et al., 2011). In addition, downregulation of multiple *KRP*s (Anzola et al., 2010) and a combination of multiple knockout KRP genes also contributed to an increase of CDK activity in *Arabidopsis* that stimulated cell proliferation. This misregulation of the cell cycle in the mutant *krp* galls most likely contributed to the accumulation of CDKA;1 in roots (Cheng et al., 2013), causing the super-activation of CDK/CYC complexes in the absence of single (Boruc et al., 2010), or multiple *KRP* members, and thus allowing a faster entry into the mitotic phase during RKN interaction. Despite the enhanced mitotic activity observed in the *krp2* and *krp2^-/-^/krp6^-/-^* mutants, GC matured normally, without compromising nematode viability (Vieira et al., 2013).

Mild defects were observed in *krp5* knockout lines, mainly at later time points (14 and 21 DAI), as GC induced on these mutants contained less cytoplasm and larger vacuoles in comparison to their respective wild-type (Coelho et al., 2017). Likewise, *KRP5* deficiency revealed only very mild defects in *Arabidopsis* plants, yet, showed some negative effect on the cortical cell size of etiolated hypocotyls (Jégu et al., 2013). Lack of KRP6 led to some extent to the inhibition of the mitotic activity within the GC, as these were significantly smaller than in the wild-type (Vieira et al., 2014). Interestingly, lack of KRP6 resulted in the presence of more cell wall stubs between the nuclei in a fraction of these GC, indicating the potential role of KRP6 in cytokinesis (Vieira et al., 2014). Single mutation in the *krp* genes caused minimal effects on plant phenotype, probably due to their partial functional redundancy (Cheng et al., 2013). Our results showed that the absence of KRP5 and KRP6 in cells with an amplified cell cycle status like the GC can have subtle deficiencies in their phenotype during gall development, as nematodes associated with *krp5* and *krp6* lines showed a delayed development and reduced offspring (Vieira et al., 2014; Coelho et al., 2017).

## Artificial Expression of *Krp* Genes Induces Dramatic Changes in Gall Morphology

**FIGURE 1:**
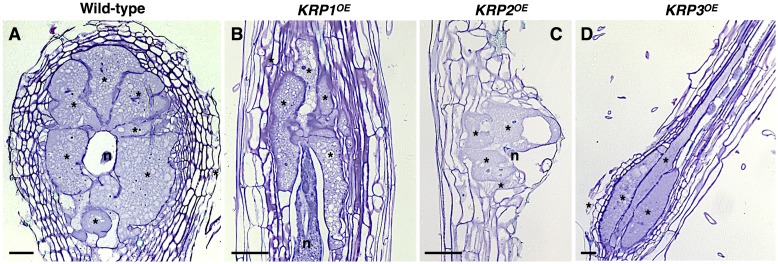
Functional analyses of the *Arabidopsis* Kip-Related Protein (KRP) gene family in galls induced by the root-knot nematode *Meloidogyne incognita* show a strong inhibition of mitosis in feeding sites leading to a drastic decrease in gall size. **(A–D)** Examples of the morphological changes observed throughout the ectopic expression of *KRP1* **(B)**, *KRP2* **(C)** and *KRP3* **(D)** compared to wild-type **(A)**. An overall reduction of gall size was observed for the *KRP^OE^* lines as a consequence of blockage of mitotic activity in giant cells as well as in neighboring cells. A noteworthy change in giant cell morphology was observed for *KRP3^OE^* line, resulting in elongated giant cells with reduced size. Asterisk, giant cell; n, nematode. Scale bars = 50 μm.

Several studies have demonstrated that *KRP*s are crucial negative regulators of the cell cycle (Wang et al., 1997; De Veylder et al., 2001; Liu et al., 2008; Jun et al., 2013). However, *in vitro* inhibition of kinase activity of the CDKA complex by KRPs is variable (Wang et al., 1997), and recent findings suggest that they might be implicated in a broader range of biological roles (Jégu et al., 2013; Vieira et al., 2014). In order to study the ectopic effects of each *KRP* gene (hereby named as *KRP1^OE^* to *KRP7^OE^*) in RKN-induced galls, we used a set of microscopy techniques, involving both semi-thin (**Figure 1**) and thick sectioning to whole-mount analyses of galls at different time points after nematode infection. One of the most prominent features of all *KRP^OE^* lines (with exception of *KRP6^OE^*) was a visual reduction of the NC asymmetrically surrounding GC, and the reduced size of the GC (**Figure 1**). Although inhibition levels were variable among the *KRP* genes, most galls displayed an overall reduced size compared to their wild-type counterpart. Another common feature of ectopic *KRP* levels (with exception of *KRP6^OE^*) was the significant reduction in the number of the GC nuclei associated with a severe blockage of mitotic activity (Vieira et al., 2012, 2013; Coelho et al., 2017).

The importance of polyploidization for gall expansion and maturation has been also demonstrated in plants with impaired endocycle machinery (de Almeida Engler et al., 2012). Endoreduplication occurs in a myriad of organisms and constitutes an effective strategy for cell growth. It is often found in differentiated cells with high metabolic activity (Edgar and Orr-Weaver, 2001) and is used extensively in tissues reserved for nutrient uptake and storage (Lee et al., 2009). In addition, the association between DNA content and cell expansion is regarded as a key process for understanding the mechanism of cell expansion (Kondorosi and Kondorosi, 2004; Breuer et al., 2010). After the mitotic to endocycle transition, progression through the endocycle is modulated by a subset of the same molecular components that control progression through the mitotic cell cycle. These molecular components form a complex regulatory network that produce oscillations in the activity of CDKs responsible for control DNA synthesis, resulting in alternating S and G phases leading to polyploidy (Lee et al., 2009; De Veylder et al., 2011). Therefore, it is important to point out that when ectopically expressed, *KRP*s inhibit the endoreduplication cycle, as shown by the decreased ploidy levels in *Arabidopsis* leaves (e.g., KRP2, KRP6) (De Veylder et al., 2001; Liu et al., 2008). The ectopic expression of *KRP*s also negatively affected the endoreduplication in gall cells, and it was perceived by the reduced ploidy levels for the *KRP2^OE^* and *KRP6^OE^* infected roots (Vieira et al., 2014).

Among all members of the *KRP* family, ectopic expression of *KRP2* revealed the most conspicuous reduction in size and structure of galls (Vieira et al., 2013). KRP2 has been implicated in the modulation of both mitosis and endoreduplication mechanisms, consistent with its ability to inhibit S-phase CDK activity (Verkest et al., 2005a). Similarly, in RKN-induced galls the effects caused by the ectopic *KRP2* expression can be conceptually divided in two phases: a strong *KRP2^OE^* expression at early stages of gall development that correlated with decreased nuclear divisions in GC and reduced mitosis in NC. At later stages KRP2 accumulation above their normal threshold levels will arrest endoreduplication of the GC nuclei (Vieira et al., 2013). Based on its regulatory action, KRP2 might have a stronger affinity to enable blocking the CDK/CYC complexes in galls that are necessary for driving the characteristic feeding site induced by RKN.

In a similar trend, artificial *KRP1*, *KRP3*, *KRP4* and *KRP7* expression prompted inhibition of mitosis, and this led to reduced gall sizes, particularly in the overexpressing *KRP1^OE^* and *KRP7^OE^* lines (Vieira et al., 2012, 2013; Coelho et al., 2017). A noteworthy change in GC morphology was observed in *KRP3^OE^* galls, as an ectopic *KRP3* expression caused an elongated GC phenotype with reduced cell size (Coelho et al., 2017). Increased levels of *KRP3* have been correlated with an alteration in the architecture of the shoot apical meristem, including a reduced dome size and slightly changed cell morphology (Jun et al., 2013). The regulatory mechanisms that control gall organization and GC shape involving the cell cycle have not been reported, but the results obtained after ectopic expression of *KRP3* could imply an effect of this gene on cell morphogenesis as well.

In the case of *KRP5^OE^* a less severe effect on the gall structure was observed in comparison to *KRP1*, *KRP3*, *KRP4*, and *KRP7* overexpression lines. Cell cycle inhibition in the *KRP5^OE^* line was sufficient to cause an overall reduction of the gall size, even though KRP5 had less of an effect on cell proliferation when compared to the other *KRP*s. Although KRP5 is known as a positive regulator of endoreduplication in particular plant tissues (e.g., etiolated seedlings) (Jégu et al., 2013; Wen et al., 2013), stimulation of the endocycle was not observed in GCs recorded by flow cytometry analyses (Coelho et al., 2017).

The most unexpected results were obtained upon ectopic expression of *KRP6*. In contrast to the other *Arabidopsis KRP* members, mitotic activity was triggered rather than inhibited in gall tissues overexpressing *KRP6*. This unexpected effect was caused by the formation of large galls filled with proliferating NC and GC of reduced size filled with a large number of nuclei (Vieira et al., 2014). These results were strengthened by equivalent observations using *Arabidopsis* cell cultures, where overexpression of *KRP6* was correlated with an accelerated G1-to-S and G2-to-M transitions, driving cells to enter mitosis earlier than wild-type. Likewise, phenotypic analysis of cultured *Arabidopsis* cells revealed a failure in cytokinesis, with the appearance of multinucleated cells as a consequence of ectopic *KRP6* expression (Vieira et al., 2014). This outcome could be explained if KRP6 functioned through activation of CDK/CYCD complexes by promoting their assembly or stability, or their targeting to the nucleus, resulting in more efficient phosphorylation of target proteins (Vieira et al., 2014). Overall, *KRP6* expression upon RKN infection and the phenotypic resemblance between *KRP6^OE^* cell cultures and GC morphology point toward the involvement of *KRP6* in the multinucleate and acytokinetic state of GC.

## Krps With Spotted Nuclear Localization Exhibit Aberrant Nuclear Morphology

**FIGURE 2:**
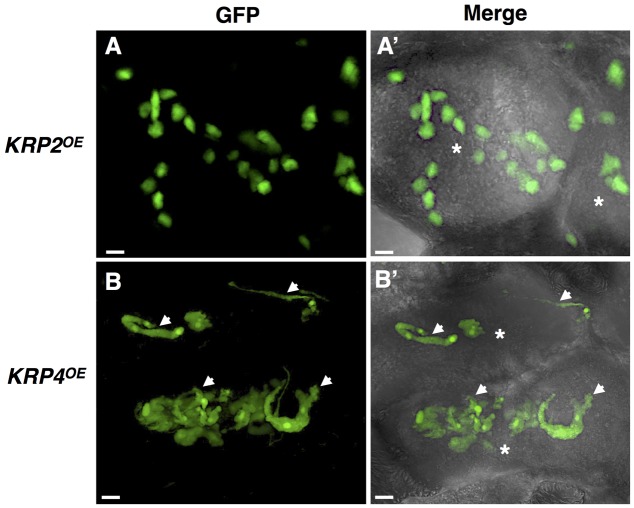
*In vivo* nuclear localization of GFP-KRP2 and GFP-KRP4 in induced galls of *Meloidogyne incognita*. Images are 3D confocal projections. (Left) Correspond to GFP fluorescence images, and (Right) Overlays of GFP fluorescence with differential interference contrast. While *KRP2^OE^* lines **(A,A’)** displayed the typical amoeboid shaped nuclei similar to the observed phenotype in wild-type giant cell, the *KRP4^OE^* lines **(B,B’)** presented aberrant elongated and apparently connected nuclei (white arrows). Asterisk, giant cell. Bars = 5 μm.

Another intriguing morphological change within mature GC was the presence of aberrant elongated and apparently connected nuclei in the *KRP1*, *KRP3*, *KRP4* and *KRP5*, overexpressing lines (**Figure 2**). In comparison, *KRP2^OE^*, *KRP6^OE^* and *KRP7^OE^* lines displayed the typical amoeboid-shaped nuclei similar to the observed phenotype in wild-type GC. Remarkably, KRP1, KRP3, KRP4 and KRP5 presented a conserved nuclear localization signal motif (YLQLRSRRL) that is responsible for the spots observed in the nuclei (Jakoby et al., 2006; Zhou et al., 2006), and these KRPs co-localized with chromosomes during different phases of mitosis. The specific role of these KRPs during mitosis was unclear, but depletion of these proteins in a precise timing associated with mitosis seemed to be imperative. For example, KRP4 degradation during sister chromatid separation appeared to be a prerequisite for normal mitosis (Boruc et al., 2010). It is conceivable that the progressive cumulative action of these KRPs during GC nuclear division leads to the aberrant, extended nuclei phenotype. The possible consequences of such KRP accumulation could involve defective DNA replication, erroneous chromosome segregation, or defects on chromosome organization within the nuclear envelope. Recent nuclear analysis of the aberrant nuclei seen in mature GC upon ectopic *KRP3* and *KRP5* expression revealed that the increased nuclear volume may not only be linked to increased ploidy levels, but also to be the result of accumulation of mitotic defects (Antonino de Souza et al., 2017).

## Deregulation of the Cell Cycle Machinery in Galls Can Inhibit Nematode Development

The RKN NFS is considered a resilient metabolic sink, showing features of specialized transfer cells with high metabolic activity and an increased cytoplasmic density. The last feature implicates the replacement of a large central vacuole by several smaller ones, an increased number and size of nuclei, proliferation of organelles and thickened cell walls with finger-like protuberance allowing for an increase in membrane surface for solute uptake (Berg et al., 2008; Rodiuc et al., 2014). As mentioned above, *KRP* overexpression caused a decrease in GC size and structure, thus reducing the capacity of acting as a sink-like cell and apparently slowing down the metabolic activity needed for gall functioning. In parallel, the reduced network of NC that surrounds the GC may exert a significant impact by causing a reduction of nutrient transfer into the GC needed for nematode development.

It is remarkable that RKN were able to induce a feeding site in all *KRP^OE^* lines, reinforcing the robust capability of the RKN to maneuver the plant cell cycle machinery in their favor. Despite the different levels of decreased gall size observed for the different *KRP^OE^* lines, these lines were able to limit the nutrient demands for RKN development and affect offspring production. Restrained nematode development was more pronounced in the *KRP1^OE^*, *KRP2^OE^* and *KRP7^OE^* lines and to a lesser extent in the remaining *KRP* members (Vieira et al., 2012, 2013, 2014; Coelho et al., 2017).

## Concluding Remarks

Former efforts have been conducted toward understanding the molecular mechanisms driving the formation and development of RKN feeding sites (Gheysen and Mitchum, 2009). The importance of a balanced cell cycle during gall formation and development has been supported by several studies (de Almeida Engler et al., 1999, 2012; Vieira et al., 2013). Functional analyses of the seven *KRP* inhibitor genes of *Arabidopsis* revealed that different family members might exert distinct functions during RKN feeding site development, although possessing an inhibitory effect in their cell cycle machinery. During gall development a level of regulation of the cell cycle by KRP2, KRP5 and KRP6 most likely takes place in order to prevent irreversible damage to the host plant root. These three *KRP*s naturally expressed in galls might fine-tune CDK activity and control the transition of the mitotic cycle to the endocycle. The control of the gall-related cell cycle machinery does not seem to involve the activation of the four remaining *KRP*s (*KRP1*, *KRP3*, *KRP4* and *KRP7*). Nevertheless, our functional studies ectopically expressing *KRP*s have shown that inhibition of the cell cycle CDK/CYC complexes compromised nematode development leading to decreases in their offspring.

A detailed analysis of the diverse members of the *KRP* multi-gene family highlighted the extent of the functional regulation of this family of CKIs during gall formation and uncovered potential novel functions for this family. The occurrence of an aberrant cell cycle in GC makes them a useful model for dissecting cell cycle gene function in galls, and as well as in plant cells. These studies provide an example of how artificial and continuous expression of *KRP* inhibitors in GCs can be deleterious for gall development. Future studies focused on engineering expression of *KRP* genes using promoters preferentially active in galls are in progress and can be considered an attractive route for RKN control. For this approach, within this family of cell cycle inhibitors, *KRP2* followed by *KRP1*, *KRP7*, *KRP3* and *KRP4* seem to be the best candidates to be considered for management of the cell cycle in galls. An analogous strategy might be envisaged for controlling the infection of other plant pathogens that exploit the host cell cycle machinery.

## Author Contributions

PV and JAE wrote the review and thank all the authors who contributed with data for all KRP manuscripts mentioned herein.

## Conflict of Interest Statement

The authors declare that the research was conducted in the absence of any commercial or financial relationships that could be construed as a potential conflict of interest.
